# The chromosome level genome assembly of the Asian green mussel, *Perna viridis*

**DOI:** 10.1038/s41597-024-03802-2

**Published:** 2024-08-28

**Authors:** Sandhya Sukumaran, V. G. Vysakh, Wilson Sebastian, A. Gopalakrishnan, Lalitha Hari Dharani, Akhilesh Pandey, Abhishek Kumar, J. K. Jena

**Affiliations:** 1 ICAR-Central Marine Fisheries Research Institute, Ernakulam North P.O., Kochi, Kerala 682018 India; 2 Nucleome Informatics Pvt. Ltd., NKC Centre for Genomics Research, 2nd Floor, 3 Cube Towers, White Field Rd, HITEC City, Hyderabad, Telangana 500081 India; 3 Institute of Bioinformatics, International Technology Park, Bangalore, Karnataka India

**Keywords:** Genome assembly algorithms, Genome assembly algorithms

## Abstract

The Asian green mussel, *Perna viridis* is an important aquaculture species in the family Mytilidae contributing substantially to molluscan aquaculture. We generated a high-quality chromosome level assembly of this species by combining PacBio single molecule sequencing technique (SMRT), Illumina paired-end sequencing, high-throughput chromosome conformation capture technique (Hi-C) and Bionano mapping. The final assembly resulted in a genome of 723.49 Mb in size with a scaffold N50 of 49.74 Mb with 99% anchored into 15 chromosomes. A total of 49654 protein-coding genes were predicted from the genome. The presence of 634 genes associated with the cancer pathway and 408 genes associated with viral carcinogenesis indicates the potential of this species to be used as a model for cancer studies. The chromosome-level assembly of this species is also a valuable resource for further genomic selection and selective breeding for improving economically important aquaculture traits and augmenting aquaculture productivity.

## Background & Summary

Shell fishes, especially bivalve molluscs, form a major component of world culture fisheries, accounting for more than 20% of the global aquaculture production^1^, and are relished as a delicacy in many countries. Molluscs belonging to the family Mytilidae, in particular, are very important globally, contributing to 6.2% of the total mollusc aquaculture^1^. Aquaculture of many of these species is popular due to their fast growth rate, tolerance to a wide range of environmental conditions, amenability to culture conditions and ease of reproduction. The capacity to accumulate a wide range of environmental pollutants and the ability to tolerate harsh environmental conditions makes them ideal sentinel species for environmental biomonitoring. Bivalves are considered as engineers of the ecosystems^2^ as they recycle nutrients through filter feeding, clean water and protect coastlines from extreme weather conditions by forming reefs^3,4^.

*Perna viridis* or green mussel is a widely distributed species across India and the Indo-Pacific^5^ and has been reported to tolerate a wide range of temperature and salinity conditions. It is an important aquaculture species worldwide due to its ease of culture in confined conditions, rapid growth rate and tolerance to diverse environmental conditions^5^.

It contributes substantially to the bivalve fishery and is in high demand in both the domestic as well as export markets^6^. Hatchery technology for the production of seeds is also standardized making this one of the priority species in bivalve farming. The annual production of bivalves in India in 2017 was estimated at 1,03,639 tons of which mussels contributed around 22.2%^7^. Parasitic diseases (mainly due to protozoan parasites, *Perkinsus olseni* and *Perkinsus beihaiensis*) constitute a major threat to *P. viridis* aquaculture in India causing substantial mortalities in farms^8^. Genomic and transcriptomic investigations on this species are vital to understand genes, gene combinations and signaling pathways that determine economically important traits like growth, reproduction and disease resistance. In addition to aquaculture, *P. viridis* is also important as a biomonitor as it is capable of accumulating heavy metals and other environmental pollutants in large quantities^9,10^. Whole genome information is valuable for understanding the genomic pathways involved in response to pollutants.

Green mussels are nutritionally enriched with polyunsaturated fatty acids (PUFAs), essential minerals, balanced amino acids, and vitamins^11,12^. The sessile nature of bivalves has made them adaptable to local environmental stressors like variations in pH, temperature, salinity and air exposure, as well as chemical and pathogenic stressors in the water column due to filter feeding habit^13^. Constant exposure to stressors requires robust adaptation mechanisms, and the molecular basis of bivalve stress responses and gene families involved have been investigated in oysters and clams^2,14,15^. Due to the absence of an adaptive immune system in bivalves, specialized tolerance mechanisms have been developed to combat constant exposure to pathogens^16^. Cellular function is maintained during infection by protein recycling pathways, chaperone proteins and apoptotic inhibitors^17,18^. The high diversity of inhibitor of apoptosis proteins (IAPs) in bivalves indicates the role of apoptosis regulation in stress tolerance^14,19^.

Neoplasms have been reported in mollusks, mainly neoplastic diseases of the hematopoietic system. Gonadal and disseminated neoplasms are widely reported in mollusks^20^. It is now considered as a transmissible disease that is transmitted between individuals through physical transfer of cancerous cells^21^ and this is referred to as bivalve transmissible neoplasia (BTN)^22^. Neoplastic diseases have been reported in bivalves belonging to the family Mytilidae^22^. The whole genome of *P. viridis* can be a valuable tool to investigate the genes involved in cancer pathways and thus *P. viridis* can be a model organism for such investigations on transmissible neoplasia.

Despite the importance of *P. viridis*, genomic resources are very few. Transcriptomic resources are available for *P. viridis* with respect to toxicity to metals, endocrine disruptors, organic pollutants and engineered nanoparticles^23^. The durability of the byssus threads of *P. viridis* has been investigated through genomics and transcriptomics^24^. The diploid chromosome number of *P. viridis* is 30 (2n) and the karyotype is composed of ten metacentric and five submetacentric chromosome pairs^25^. We estimated the genome size of *P. viridis* as 842 Mb based on flow cytometry analysis. The chromosome level genome assembly of *P. viridis* was assembled by adopting an integrated approach using PacBio Sequel II, Illumina, Hi-C Sequencing and Bionano mapping. The chromosome level genome assembly of *P. viridis* is an important genomic resource for further genomic improvements, genomic selection and selective breeding programmes for improving economically important traits like growth and disease resistance of this important aquaculture species. In addition, the genes and gene families that are important in cancer pathways can be elucidated, which can be further utilized for gaining insights into human cancers. The genes and gene families involved in tolerance to xenobiotics can also be explored to identify genomic biomarkers of toxicity.

## Methods

### Sample collection

**Fig. 1:**
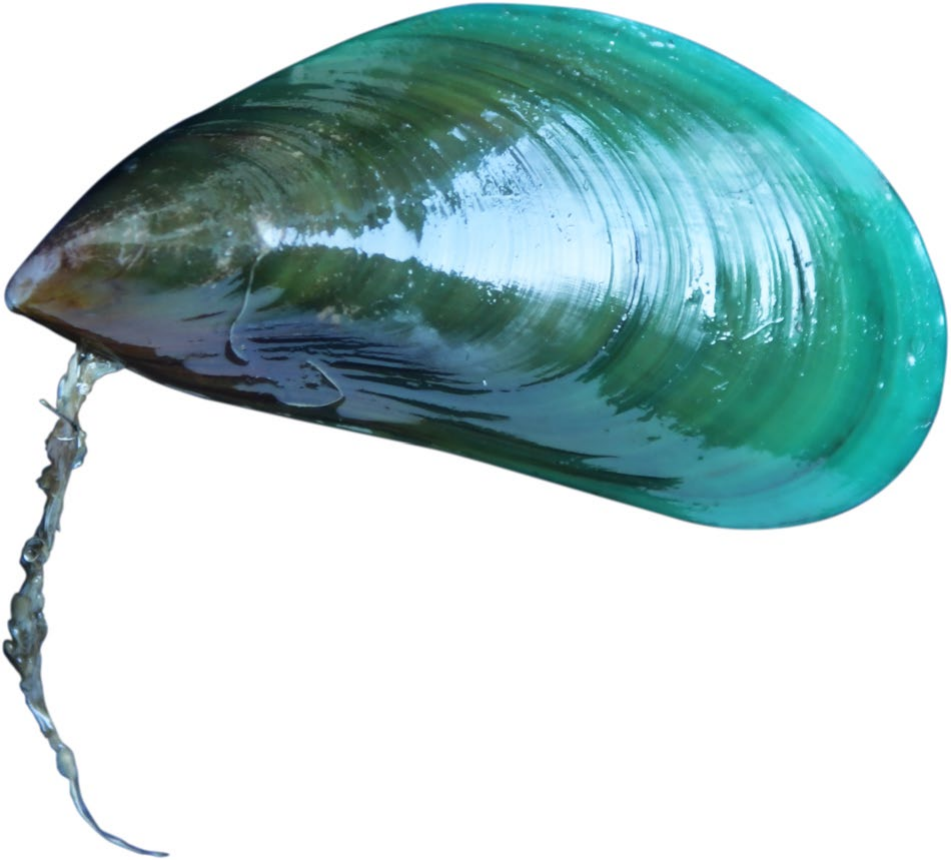
A photograph of the Asian green mussel, *Perna viridis* used for whole genome sequencing.

Male specimens of the Asian green mussel, *Perna viridis* (Fig. 1) were collected live from mussel beds off Munambam (10°10′46″N; 76°9′53″E) (Kochi, Kerala, India). The adductor muscle, mantle, gonad, foot and gill tissues were dissected out and flash frozen in liquid nitrogen and stored at −80 °C until DNA and RNA extraction and subsequent sequencing.

### DNA extraction and genome sequencing

High molecular weight genomic DNA was extracted from adductor muscle tissue using a genomic DNA isolation kit (QIAGEN 100/G) following manufacturer’s protocol. The quantity and quality of isolated DNA were measured using the NanoDrop 2000 spectrophotometer (Thermofisher Scientific, Massachusetts, USA). For short-read sequencing, paired end libraries with an insert size of 500 bp were prepared using the KAPA HyperPlus kit (Basel, Switzerland) following manufacturer’s protocol and the libraries were sequenced on the Illumina Novaseq 6000 platform. A total of 123 Gb data was generated from the Illumina short-read DNA library, with 169X genome coverage.

Long read genome sequencing was performed using the PacBio Sequel II system (Pacific Biosciences, California, USA). PacBio libraries were constructed using SMRTbell Express template preparation kit 2.0 (Pacific Biosciences, California, USA) according to manufacturer’s protocol and purified using AMpure PB beads (Pacific Biosciences, California, USA). The purified libraries were treated with SMRTbell Enzyme Cleanup Kit 2.0 to remove any unbound adapters and damaged DNA and purified again with AMPure PB beads. Purified libraries were size selected using BluePippin (Sage Science, USA). A library with an insert size of 15.935 kb was loaded onto one SMRTcell containing 8 M ZMW and sequenced in the PacBio Sequel II system in CCS/HiFi mode. A total of 71.17 Gb (98x coverage) PacBio long sequencing reads (clean data) with N50 read length of 21.933 Kb were were obtained after removing adaptors in polymerase reads. The raw data generated was assembled and annotated subsequently.

Chromosome level assembly was constructed using the Hi-C technique and the Hi-C libraries were constructed as reported previously^26,27^. Adductor muscle tissue cells were fixed with formaldehyde to preserve the 3D structure of DNA in the cells, and the cells were then digested using the Mbol restriction enzyme. The 5’ overhangs were then repaired with biotinylated residues. After ligation of the blunt-ended fragments *in situ*, the isolated DNA was reverse-cross linked, purified and filtered to remove biotin-containing fragments. End repair of DNA fragments, ligation of the adapter, and polymerase chain reaction (PCR) were performed successively. Library concentration was determined using the Qubit 3.0 platform and the insert size using the LabChip GX platform (PerkinElmer). Finally, the high quality Hi-C libraries were sequenced on the Illumina Novaseq 6000 platform with a strategy of 2 × 150 bp and the sequencing data were used for chromosome level assembly^28^. A total of 359 Gb of Hi-C paired-end raw reads (150 bp in length) were generated. Subsequently, fastp was applied to filter the adapters, and those reads shorter than 30 bp or of low-quality (quality scores <20) were removed. The high quality reads were subsequently used for construction of the chromosome level assembly.

Bionano optical mapping was performed with Saphyr’s streamlined workflow (Bionano Genomics) to improve genome assembly. The ultra high molecular weight DNA (UHMW DNA) extraction was performed with the Bionano Animal Tissue DNA Isolation Kit (San Diego, CA, USA), using the manufacturer’s protocol. The prepared UHMW DNA was then labelled, counterstained and imaged sequentially in nanochannels on the Bionano Saphyr instrument. Raw data were filtered if (a) the molecule is <150 Kb; (b) molecule SNR (signal to noise ratio) <2.75 & label SNR <2.75; and (c) label intensity >0.8. The filtered data were assembled and corrected using BIONANO Solve v3.4 (Bionano Genomics) and anchored to the reference genome to produce the scaffolds. A total of 15,933 maps were generated with a map N50 of 4.1 Mb. Transcriptome sequencing of the expressed genes was then performed to improve annotation.

### RNA extraction and Transcriptome sequencing

The adductor muscle, mantle, gonad, foot and gill tissues were dissected out and total RNA was extracted from each tissue using Trizol reagent (Invitrogen) and the isolated RNA was purified using Nucleospin RNA Cleanup Kit (Macherey-Nagel, Germany). Quantity of the purified RNA was measured using the Qubit 3.0 Flurometer and the purity was checked using NanoDrop 2000 (Thermofisher Scientific, Massachusetts, USA). The integrity of the sample was confirmed on a Bioanalyzer (Agilent 2100) and the RNA extracted from all the tissues was pooled at equimolar concentration. The RNA was subjected to cDNA synthesis and amplification using the NEBNext Single Cell/Low input cDNA synthesis and amplification module in conjunction with the Iso-Seq Express Oligo Kit (Pacific Biosciences, California, USA). The Pronex beads (Promega, Wisconsin, USA) were used for purification of the cDNA before amplification and later for size selection of the amplified product. The library was constructed using SMRTbell Express template preparation kit 2.0 (Pacific Biosciences, California, USA) according to the manufacturer’s protocol. The library was purified using Pronex beads (Promega, Wisconsin, USA) and the library size was assessed using Bioanalyzer (Agilent 2100). About 80 pM of the library was loaded onto one SMRT cell containing 8 M ZMW and sequenced in the PacBio Sequel II system in CCS/HiFi mode. A total of 1.8 Gb high quality reads was generated from Iso-Seq sequencing of the *P. viridis* pooled transcriptome.

### Estimation of the genome size using k-mer analysis and flow cytometry

K-mer analyses were performed using Jellyfish v.2.3.1^29^. and GenomeScope (v.2.0)^30^ with 21-mer frequencies. The genome size was estimated at 651 Mb with a heterozygosity value of 0.51.

The genome size of the Asian green mussel, *Perna viridis* was also estimated using flow cytometry. In flow cytometry, genome size is estimated by staining the DNA of individual cells using propidium iodide^31^ or DAPI^32^ and analyzing the fluorescence. Haemolymph was withdrawn from the adductor muscle of each mussel using a 1-ml syringe prefilled with 0.01 M PBS. The haemolymph was centrifuged at 5000 rpm for 8 minutes to sediment the haemocytes. The cells were subsequently washed twice with 0.01 M PBS to remove any residues and fixed with ice-cold 70% ethanol for 2 hours at 4 °C. Cells were again washed with 0.01 M PBS to remove any residual ethanol. The cells were then stained with propidium iodide and analyzed using flow cytometry. Chicken red blood cells were used as a standard. The genome size of the Asian green mussel, *Perna viridis* was estimated using a Beckman Coulter Cytoflex flow cytometer with laser excitation at 488 nm with a minimum of 10,000 events (cells) per sample. The genome size was estimated to be 842 Mb.

The estimated genome size using K-mer analyses (651 Mb) is smaller than that estimated by flow cytometry (842 Mb) and assembled by PacBio (723.49 Mb) in the present study.

### *De Novo* genome assembly

**Fig. 2:**
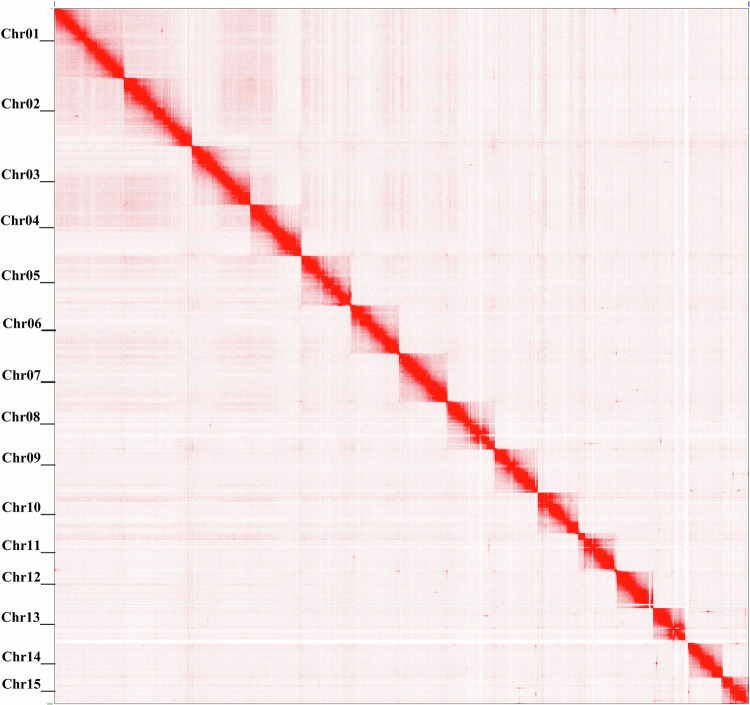
Contact map plot of the Asian green mussel, *Perna viridis* genome. The raw read pairs from Hi-C were aligned with the genome sequences. The x and y axes indicate their positions. The positions of the read pairs are indicated by the red dots. A high density of red dots indicates that they are located on the same chromosome.

**Fig. 3:**
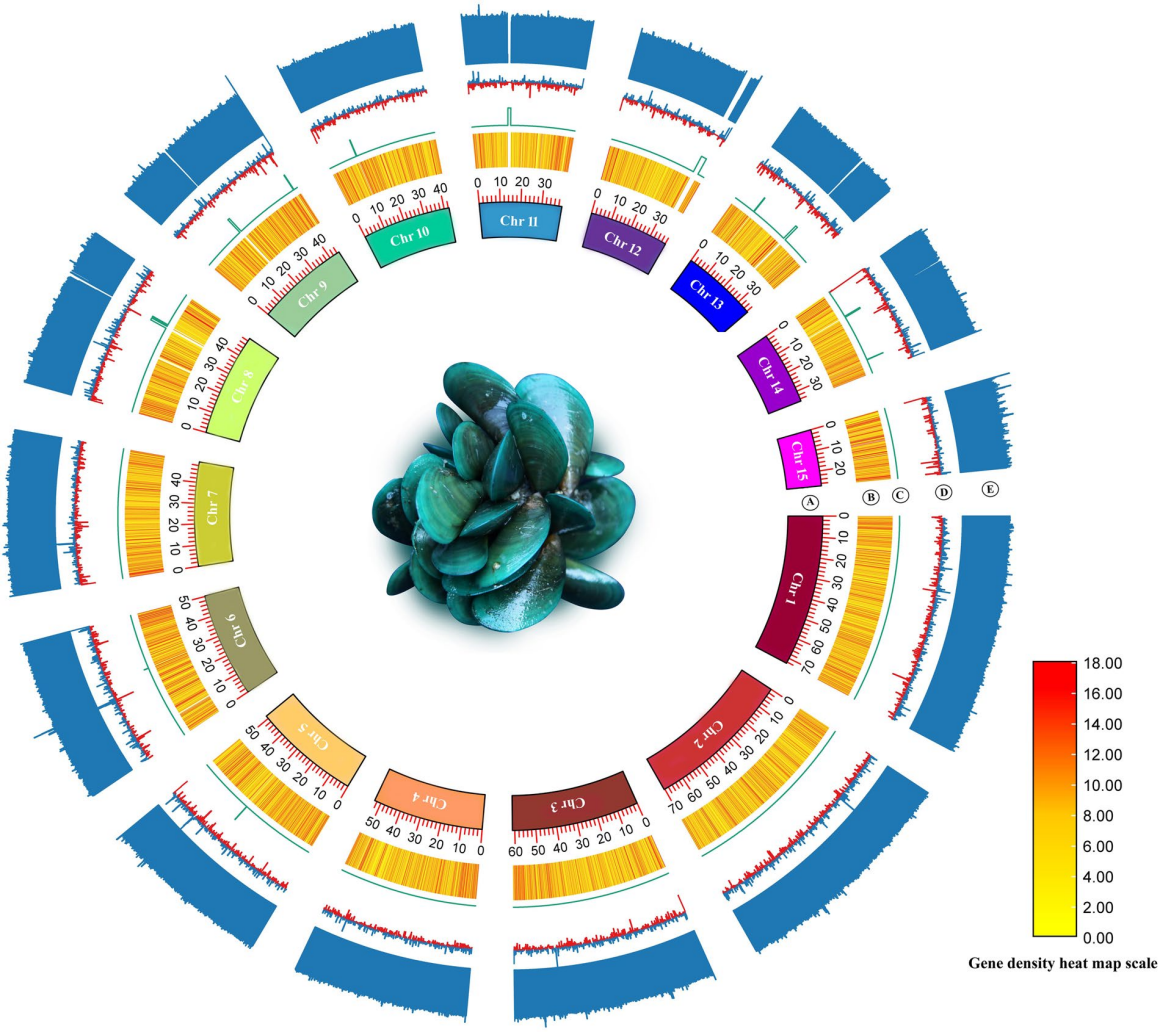
Circos plot of the *P. viridis* genome assembly. (**A**) 15 chromosomes (**B**) Gene density heat map (**C**) N -ratio, (**D**) GC skew (**E**) the distribution of GC content.

Illumina sequencing data was used to polish preliminary contigs. The Illumina reads were filtered using fastp software (v.0.23.1)^33^ for filtering adapter sequences and low-quality reads and the remaining reads were used for polishing of preliminary contigs. The preliminary contig assembly was generated using PacBio sequencing data. The subreads generated by the PacBio sequel II system were used to call the ccs reads using the SMRT link v10.2. Multiple subreads of the same SMRTbell molecule were combined using a statistical model to generate one highly accurate consensus sequence (CCS), also called a HiFi read, along with base quality values. Further, contig-level assembly was performed using the Hifiasm (v0.16.1)^34^. Hifiasm is an efficient and fast haplotype-resolved *de novo* assembler specifically for PacBio HiFi reads. Sequences were corrected using Hifiasm. Further, the assembly was corrected by haplotype-aware read and phased string graph construction. Partially phased assemblies of high quality were generated. The primary contigs were then polished with Illumina reads using Pilon v.1.2^35^. Scaffolding was performed using Bionano data, integrating long-range structural information from Bionano maps with the assembled contigs. The gaps between contigs and misassemblies were resolved and the contigs were oriented along the chromosomes. The structural data from Bionano highlighted the connections between contigs that are distant from each other in the linear genome but physically close to each other in the three-dimensional chromatin structure using BIONANO Solve v3.4 (Bionano Genomics). Hybrid scaffolds were generated as the output. The hybrid scaffolds were again super-scaffolded with Hi-C reads using scaffHiC v1.1 (https://github.com/wtsi-hpag/scaffHiC). The Hi-C chromosome contact map plot is shown in Fig. 2. Large scaffolds were constructed using graph construction and link scoring function, and the best final scaffolds were selected. Manual curation was carried out using the tool “pretext” to get the chromosomal level assembly. The final assembly resulted in a genome of 723.49 Mb in size with a scaffold N50 of 49.74 Mb with 99% anchored into 15 chromosomes. The circus plot of the genome assembly is shown in Fig. 3. The assembly statistics are given in Table 1. The completeness of the assembly was evaluated using BUSCO assessment with BUSCO v5.3.2^36^. A total of 924 out of the 954 (96.85%) of the Metazoa gene set (Metazoa_Odb10) were fully identified in the assembled genome. The genome module benchmark values were calculated as C: 96.85%, including [S: 96.0%, D: 0.85%], F: 1.8%, M: 1.4% and n = 954 (C: complete, S: single-copy, D: duplicated, F: fragmented, M: missing and n: total BUSCO groups of Mollusca Odb10 data). A total of 4594 out of the 5295 (86.76%) of Mollusca gene set (Mollusca_Odb10) were fully identified in the genome. The genome module benchmark values were calculated as C: 86.7%, including [S: 86.0%, D: 0.7%], F: 3.3%, M: 10.0% and n = 5295 (C: complete, S: single-copy, D: duplicated, F: fragmented, M: missing and n: total BUSCO groups of Mollusca Odb10 data). The BUSCO values assessed using Metazoan dataset (single copy and duplicated) is higher than the high quality genome assembly of the hard shelled mussel, *Mytilus coruscus* (91.09%)^37^, the manila clam, *Ruditapes philippinarum* (91.0%)^38^ and the pearl oyster, *Pinctada fucata* (95.2%)^39^ indicating the high quality of the assembly of *P. viridis* in the present study. Transcriptome assembly was then performed to improve genome annotation.

**Table 1:** Statistics of the assembled genome of Asian green mussel, *Perna viridis*.

Genome assembly statistics	Data	
Total Length	723496279 bp	
No: of super scaffolds/contigs assigned to chromosomes	15	
No: of unanchored contigs	31	
Largest contig	72616575 bp	
GC rate (% of genome)	32.9%	
N50 Scaffold length	49738958 bp	
N90 Scaffold length	36560911 bp	
Repeat elements (% of genome)	44.82%	
**BUSCO genome completeness score**	**Data**	**Ratio**
Complete BUSCOs	923	96.8%
Complete and single copy BUSCOs (C)	916	96.0%
Complete and duplicated BUSCOs (D)	7	0.80%
Fragmented BUSCOs (F)	17	1.80%
Missing BUSCOs	14	1.40%
**Total number of Metazoa orthologs**	**954**	

### *De Novo* transcriptome assembly

The iso-seq data were processed by calling the circular consensus sequence (CCS) using SMRT tool ‘ccs’ and HiFi reads were generated. The HiFi reads were subjected to refinement and clustering. Full length non-chimeric reads (FLNC) were generated using the tool ‘isoseq. 3 refine’ (https://github.com/nf-core/modules/tree/master/modules/nf-core/isoseq. 3/refine), which removed poly(A) tail and concatemer from the reads. The trimmed full-length reads were clustered at the isoform level and consensus is called. The transcripts with a predicted accuracy ≥0.99 are considered as the high quality reads and <0.99 are considered as low quality reads. The redundant transcripts generated by reads which originated from 5’ degraded RNA were removed by collapsing. The high quality reads were mapped to the reference genome of *P. viridis*, using pbmm2 tool (https://github.com/PacificBiosciences/pbmm2) and these mapped reads were then collapsed using the’isoseq. 3 collapse’ tool. Subsequently, unique isoforms were generated in GFF format along with secondary files containing information about the number of reads supporting each unique isoform. We found alignment coverage (alignment length to transcript length) of 100% for expressed genes in the genome assembly. The genome was further analyzed for repeat content and type of repeat units.

### Repeat annotation

Annotation of repeat units was performed using *ab initio* prediction and homology annotation. RepeatModeler (http:// https://www.repeatmasker.org/RepeatModeler)^40^, Repeat Scout^41^ and LTR FINDER^42^ were used to identify various types of repeat elements. RepeatMasker (https://www.repeatmasker.org/)^43^ was employed to construct a repeat elements library based on the Repbase TE v22.11 database^44^. The Tandem Repeats Finder was used to identify the Tandem elements. Known repeat element types were identified from the Repbase database using Repeat Masker and Repeat ProteinMask. A total of 324.3 Mb of repetitive elements were identified in the genome of the Asian green mussel, accounting for 44.82% of the genome.

The repeat content is lower than that of the Korean mussel, *Mytilus coruscus* (52.83%)^37^ the Philippine horse mussel, *Modiolus philippinarum* (62.0%)^45^ and the deep-sea mussel, *Bathymodiolus platifrons* (47.9%)^45^. The statistics of the repeat elements of the *P. viridis* genome are shown in Table 2. Further, protein coding gene prediction was performed on repeat masked assembly.

**Table 2:** Statistics of repeat elements in the genome of *Perna viridis*.

Repeat classes	Length	Percentage of genome
Retro elements	70340305 bp	9.72%
DNA transposons	6201062 bp	0.86%
Unclassified	240875560 bp	33.29%
**Total interspersed repeats**	**317416927 bp**	**43.87%**
Simple repeats	5382517 bp	0.74%
Low complexity	1509814 bp	0.21%
**Total**	**324309258 bp**	**44.82%**

### Protein coding gene prediction and functional annotation

Gene predictions were undertaken using ab initio, homology-based and transcriptome-based prediction strategies. The predictions were made using the AUGUSTUS gene prediction server (https://bioinf.uni-greifswald.de/augustus/), as implemented in the OmicsBox version 2.2 platform (https://www.biobam.com/omicsbox/) using the repeat masked sequences as input with ab initio and extrinsic evidence options. Homology based predictions were performed using the proteome data of *Bathymodiolus platifrons*, *Crassostrea gigas*, *Crassostrea viriginica* and *Modiolus philippinarum* retrieved from the Mollusc DB database (http://mgbase.qnlm.ac/home)^46^. A final non-redundant gene set was created by merging all the gene sets from these three approaches using BRAKER^47^. The combined gene set generated through all the prediction strategies was functionally annotated via OmicsBox using biological databases; Uniprot (https://www.uniprot.org/), KEGG pathways and EggNOG databases^48^. The InterProScan program was used to perform gene ontology annotations. A total of 49654 protein-coding genes were predicted with a mean length of 1081 bp. About 46304 (93.25%) of the total predicted genes were assigned with function annotation. This high number of protein coding genes is comparable to other molluscs like *Mytilus galloprovincialis* (Family: Mytilidae) (core set of 45000 genes)^49^ and *Ruditapes philippinarum* (Family: Veneridae) (set of 40909 genes) (Mollusc DB: http://mgbase.qnlm.ac/home). KEGG analysis revealed 634 genes associated with the cancer pathway and 408 genes associated with viral carcinogenesis. We also predicted 4604 long non-coding RNAs from the genome using the programme PLEK^50^. The annotated data was further used for ortholog and phylogenetic analyses.

### Ortholog and phylogenetic analyses

**Fig. 4:**
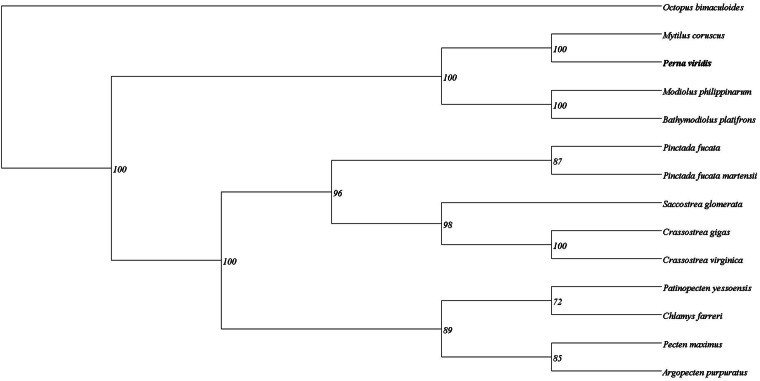
Maximum likelihood phylogenetic tree generated using single copy orthologous genes from 12 representative Molluscan species and *Perna viridis*. *Octopus bimaculoides* was used as an out group. The tree was generated using IQ-TREE v 2.1.4.

We downloaded reference protein sequences of 12 representative species, including the hard shelled mussel or Korean mussel *Mytilus coruscus*, the Philippine horse mussel *Modiolus philippinarum*, the deep sea mussel *Bathymodiolus platifrons*, the Akoya pearl oyster *Pinctada fucata* and *P. fucata martensii*, the Sydney rock oyster *Saccostrea glomerata*, the Pacific oyster *Crassostrea gigas*, the eastern oyster *Crassostrea virginica*, the Yesso scallop *Patinopecten yessoensis*, the Zhilong scallop *Chlamys farreri*, the Great scallop *Pecten maximus* and the Peruvian calico scallop *Argopecten purpuratus* from the Mollusc DB database (http://mgbase.qnlm.ac/home)^46^. Subsequently the protein sets were filtered by removing protein sequences with less than 50 amino acids. The orthologous genes were identified from this sequence dataset (including *Perna viridis* protein set) using OrthoFinder v 2.5.4 (-S diamond -I 1.5 -M msa -A maf -T fasttree -oa)^51^. Single copy orthologous genes from all species (620 numbers) were aligned and concatenated and phylogenetic analyzes were performed. A maximum Likelihood (ML) tree was constructed based on these alignments using IQ-TREE v 2.1.4 (–seqtype AA -m JTT + F + I + G4 -bb 10000 -alrt 10000)^52^ (Fig. 4). *P. viridis* clustered with the species belonging to the family Mytilidae, *Mytilus coruscus*, corroborating the findings from traditional taxonomy.

## Data Records

The genome assembly of *P. viridis* has been deposited with NCBI, GenBank under accession number JAVAIJ000000000^53^. BioProject ID: PRJNA964485 and BioSample ID: SAMN34473462. The transcriptome sequence dataset has been deposited in the NCBI Sequence Read Archive (SRA) under the project number SRR26189005^54^. The DNA sequence dataset generated from Illumina Novaseq 6000 platform (paired end library) has been deposited under project number SRR24363657^55^. The DNA sequence dataset generated from the PacBio Sequel II platform has been deposited under the project number SRR26114374^56^. The DNA sequence dataset generated from Hi-C sequencing (Illumina) has been deposited under the project number SRR26132871^57^. Bionano maps generated from Bionano Saphyr were deposited as supplementary file SUPPF_0000005531. The files of the assembled genome and annotaton of *P. viridis* have been deposited in the Figshare database ^58^.

## Technical Validation

The quality of extracted DNA was analyzed using the agarose gel electrophoresis. The main band was around 20 kb with DNA spectrophotometer ratios (260/280) more than 1.8. Quantity of the purified DNA was measured using Qubit 3.0 fluorometer. DNA shearing was performed on Megarupture 3 system (Diagenode, Belgium) at speed settings of 31 and 32 with disposable shearing syringe. The library was constructed using the SMRTbell Express template preparation kit 2.0 (Pacific Biosciences, California, USA) as per manufacturer’s protocol. The library was purified using AMPure PB beads ((Pacific Biosciences, California, USA). The purified libraries were treated with SMRTbell Enzyme cleanup kit 2.0 to remove any unbound adapters and damaged DNA. The libraries were again purified using AMPure PB reads after enzyme cleanup. Purified libraries were size selected using BluePippin (Sage Science, USA) (10kb-50kb mode) with 0.75% DF Marker S1 High pass Cassette. Size selected SMRT libraries were purified and then subjected to primer annealing and polymerase binding using Sequel II binding kit 2.2 to prepare bound complex. About 90pM of the library was loaded onto one SMRTcell containing 8 M ZMW and sequenced in PacBio Sequel II system in CCS/HiFi Mode. The quality of the purified RNA molecules was determined by Nanodrop 2000 spectrophotometer (Thermofisher Scientific, Massachusetts, USA) as absorbance values > 1.7 at 260 nm/280 nm. The integrity of RNA was evaluated on Agilent 2100 Bioanalyzer (Agilent Technologies, California, USA) as the RIN of 8.0. We further evaluated the completeness of the P. viridis genome assembly using BUSCO v5.2.2. and 96.8% of the BUSCO genes were complete.

## Data Availability

The genome and transcriptome analyses were performed following the manuals and protocols of the cited bioinformatic software. No new codes were written for this study.
